# Determinants of overburdening among informal carers: a systematic review

**DOI:** 10.1186/s12877-020-01708-3

**Published:** 2020-08-26

**Authors:** Nienke Lindt, Jantien van Berkel, Bob C. Mulder

**Affiliations:** 1 Strategic Communication Group, Wageningen University, Hollandseweg 1, 6706 KN Wageningen, The Netherlands; 2 Consumption and Healthy Lifestyles, Wageningen University, Hollandseweg 1, 6706 KN Wageningen, The Netherlands; 3 Strategic Communication Group, Wageningen University, Hollandseweg 1, 6706 KN, P.O. Box 8130, 6700 EW Wageningen, The Netherlands

**Keywords:** Informal care, Stress, Burden, Antecedents, Adapted stress model

## Abstract

**Background:**

The world’s population is ageing, resulting in rising care demands and healthcare costs, which in turn lead to a shift from formal to informal care. However, not only is the number of potential informal carers fast decreasing, but also informal caregivers are experiencing a higher caregiver burden. This literature review aims to synthesize the literature on the common determinants of caregiver burden in Western countries, to help ensure future continuation of informal care in the home context, and to improve or sustain the quality of life of caregivers and patients alike.

**Method:**

A systematic review of peer-reviewed articles included in PubMed, Scopus, and/or PsychInfo was conducted.

**Results:**

Seventeen articles were included. The most important predictors were the duration of caregiving and the patient’s dependency level, in terms of both physical and mental dependency stemming from decreased cognitive capacity or behavioural problems. Some specific illnesses and role conflicts or captivity also increased caregiver burden, whereas social support lowered it. Being a female caregiver or having an adult–child relationship led to a higher burden.

**Conclusions:**

The most important predictors of caregiver burden are the duration of caregiving and the patient’s dependency level. In addition, the patient’s behavioural problems and cognitive capacity determine dependency level, and thus care burden. Interventions to relieve burden need to be adapted to the illness trajectory of specific diseases and corresponding needs for social support for both the recipient and the caregiver. Changing role expectations, leading to men being more involved, could reduce the disproportionately high burden for women.

## Background

The world’s population is ageing, and the percentage of older people will continue to rise in the coming years. Whereas the percentage of people aged 65 or higher was 9.1% among the world’s population in 2019, it is estimated that in 2050 older people will account for 15.9%. In Europe and Northern America, by 2050 one in four persons could be aged 65 years or over. Moreover, within that same timeframe, the number of people aged 80 years or over is expected to triple from 143 million in 2019 to 426 million worldwide [1]. Because of the ageing population, the demand for care is also rising. It has been established that, as people age, they suffer from more (chronic) illnesses, needing long-term care. Among people aged 65 years or older, 70% suffer from a chronic disease. In the population aged 75 years or older suffering from a chronic disease, 63% have two or more chronic illnesses [2]. This results in the care volume increasing by 4% every year in the time period 2000–2010, leading to higher overall healthcare costs [3].

### Informal care

In order to face these rising care demands and healthcare costs, Western governments are steering away from formal care and towards informal care. Consequently, older people are encouraged to stay at home longer instead of moving into nursing homes. In order to make this feasible, such people will have to rely on their direct network of family and friends to ensure informal care [4]. Given the trend of an ageing society and policy reforms, the need for informal care will increase in the coming years.

But how can informal care be defined? Informal care consists of all aid to a person in need of care from someone in his or her direct environment. This also entails less intensive help, help towards members of the household, and help towards institutional residents. Informal care goes further than so-called regular help. Regular help – the aid that may reasonably be expected towards members of the household – means, for instance, the care for children. Examples of informal care activities are emotional support, administrative help, guidance in arranging appointments, transport, domestic and personal care [5]. It should be stressed that informal care is unpaid, results from social rather than professional relations, and entails long-term care for sick family members or friends [6].

In 2014, more than a third of the European population (34.3%) gave some type of informal care. Most informal carers are aged between 50 and 75 and take care of partners or parents (in law) [7]. The largest group of people in need of informal care are those aged 75 and over [8].

Even though the need for informal carers is rising, current research reports that the potential support ratio – defined as the number of people of working age (25 to 64 years) per person aged 65 years or over – is rapidly decreasing. By 2050, it is estimated that, for 48 Western countries, the potential support ratio will have more than halved to below two; this means fewer than two potential informal carers per elderly person [1].

### Caregiver burden

Besides the pressing future shortage of informal carers, Dutch research has shown that almost one in 10 informal carers feels overloaded with the care demand. Even though research also stresses the dual nature of informal caregiving, thus having both negative and positive aspects (e.g. [9, 10]), one in four providing long-term and intensive informal care feels stressed out. Not only do they feel as if the care demand is never lifted from their shoulders, but also they struggle with the upkeep of their own household and thus become overstrained [11]. These figures are alarming, especially as the Dutch healthcare system and its formal care services are advanced relative to other countries.

Caregiver burden can be defined [12] as *‘a multidimensional response to the negative appraisal and perceived stress resulting from taking care of an ill individual’* (p. 846). Overburdening threatens both the physical and the psychological health of caregivers. It has been shown that overstrained caregivers use more healthcare services and prescribed medication than non-caregivers, indicating a decline in physical health [13]. Moreover, stressed caregivers report more feelings of depression, perceived lack of coping mechanisms, and concerns about their poor quality of life [14]. If this burden is combined with the decreasing number of potential informal carers, it becomes apparent that the caregiver burden needs to be alleviated in order to cope with the future rising demand for informal care.

### Research objective

Research has already been undertaken regarding the determinants of informal caregiver burden. However, most literature reviews have focused on homogeneous diagnostic groups. For instance, a systematic review that focused on caregiver burden in dementia [15] led to several categories of both patient and caregiver determinants, such as the patient’s need for support, (cognitive) function disorders, and the caregiver’s social functioning, self-efficacy, and coping traits. Other reviews have focused on caring for elderly patients with cancer [16], amyotrophic lateral sclerosis [17], Parkinson’s disease [18], and mental disorders including bipolar disorder [19] and eating disorders [20]. Caregiver burden may indeed vary across diseases; however, exclusively focusing on specific diseases limits the identification of common determinants of caregiver burden across diseases [21].

The lack of knowledge on common determinants may hamper the development of effective policies and interventions that aim to relieve caregiver burden. Current interventions generally focus on providing caregivers with both direct and indirect support, such as emotional support, advice on coping, and respite services to reduce the amount of care burden [22]. However, systematic reviews on such interventions show that the effects on caregiver burden are very small or even insignificant [23, 24].

To inform future policy and intervention development, the aim of the present review is to synthesise the literature on the common determinants of caregiver burden in Western countries, to ultimately ensure future continuation of informal care in the home context, and to improve or sustain the quality of life of caregivers and patients alike. The literature review aims to answer the following research question: *What are the determinants of caregiver burden among informal carers?*

### Hypotheses

Given its multidimensionality, the caregiver burden construct has many different definitions. According to George and Gwyther [25], it entails physical, psychological, emotional, financial, and social stressors that individuals experience because of providing care. Depending on the scope of the research and included facets, the caregiver burden definition differs per study [26].

Moreover, the distinction between objective and subjective burden adds to the complexity of the caregiver burden construct. Montgomery, Gonyea, and Hooyman [27] state that: *‘objective burden is defined as the extent of disruptions or changes in various aspects of the caregivers’ life and household. Subjective burden is defined as the caregivers’ attitudes toward or emotional reactions to the caregiving experience’* (p. 21). Thus, objective burden entails overt aspects, such as time spent on caregiving and the nature and number of tasks, whereas subjective burden encompasses caregiver perceptions of the care demands and their consequences, such as negative mood states and anxiety.

**Fig. 1:**
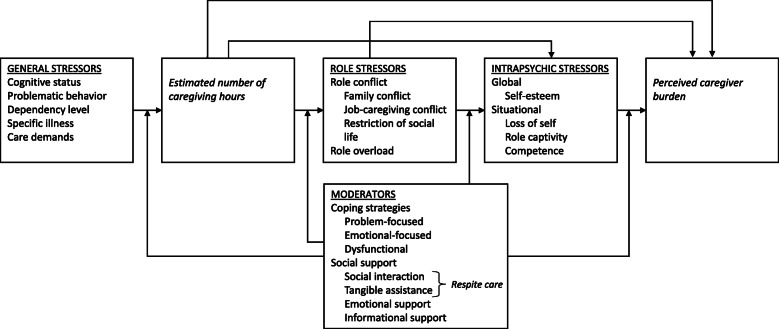
Adapted Stress Model

The multidimensionality of, and the objective–subjective distinction within, caregiver burden are both reflected in the Adapted Stress Model (ASM). The ASM describes the relationships between various categories of determinants that ultimately cause perceived caregiver burden (Fig. 1). Causality between the different constructs is implied from the left side of the ASM to the right, i.e. stressors positively affect caregiver burden, meaning that caregivers experience more stress than non-caregivers.

The ASM is based on stress theories, notably the transactional model of stress and coping [28] and the stress process model [29], as well as role theory [30].

Going from the left side of the model to the right, the ASM allows for the formulation of specific hypotheses, including their rationale.

#### H1: direct effects of stressors on perceived caregiver burden

Whereas H2, H3, and H4 describe indirect (i.e. mediated) effects of general stressors through role and intrapsychic stressors, it is hypothesised that each class of stressor in the ASM may also directly result in perceived caregiver burden.

#### H2: general stressors result in an estimated number of hours spent on caregiving

The general stressors are the patient’s cognitive status, problematic behaviour, dependency level, specific illness, and care demands. Together, these five general stressors lead to the caregiver’s *primary appraisal* of the severity of the stressor [28], which translates into the estimated number of hours that need to be spent on caregiving.

#### H3: the estimated number of caregiving hours leads to role conflict and role overload

As a result of the estimated number of caregiving hours, caregivers may experience role conflict and role overload as role stressors. Role conflicts occur when the expectations of the various roles fulfilled by the caregiver become incompatible [30]. Theoretically, this is in line with *secondary appraisal* in the transactional model of stress and coping, which is the appraisal of one’s resources (e.g. time) to manage the stressor [28]. In the case of informal care, the informal carer role may lead to conflict with the roles of being a parent or being an employee.

Role overload happens when people lack sufficient resources and time to fulfil all obligations linked to their roles [31]. For instance, an increase in the number of caregiving hours may result in role overload when the caregiver feels he or she lacks sufficient hours in the day to complete all tasks associated with every role he or she occupies.

#### H4: role stressors may lead to intrapsychic stressors

Role conflict and overload may result in role captivity, the feeling of entrapment within the caregiving role and the related experience of self-loss [32]. For instance, caregivers may give up activities related to other roles, such as quality time with their partner, in order to take care of their ill parent. It has been established that role captivity and loss of self can lead to higher feelings of depression and burden [32, 33]. In addition to this situational burden, role and intrapsychic stressors may negatively affect self-esteem and feelings of competence.

#### H5: coping strategies and social support moderate the relationships between stressors and perceived caregiver burden

Finally, the relationships between stressors and perceived caregiver burden may be moderated by coping strategies and perceived social support. Coping can be defined as the cognitive, emotional, and behavioural efforts to manage the internal and the environmental demands that challenge or exceed someone’s resources [28]. According to Carver, Scheier, and Weintraub [34], coping efforts can be divided into three categories: problem-focused coping, emotional-focused coping, and dysfunctional coping. Problem-focused coping entails strategies used to solve certain problems, such as thinking about the steps that need to be taken in order to solve the problem. When the caregiver tries to reduce or eliminate negative feelings connected to the role, such as through seeking distraction, this can be considered a type of emotional-focused coping. Lastly, dysfunctional coping happens when people do not accept the problem at hand and try to ignore or even deny reality, and this is positively associated with feelings of anxiety, depression, and perceived burden [35, 36]. In contrast, problem-focused coping does not appear to be related to caregiver burden [36, 37]. Whether emotional-focused coping and burden are related is not consistent in the current literature. However, cases of informal care among palliative cancer patients or people suffering from dementia show that emotional-focused coping leads to a decrease in perceived burden [37, 38].

Perceived social support is the second moderator in the ASM. In general, informal caregivers receiving social support from their own contacts or professionals experience less burden than others [39]. According to Thompson, Futterman, Gallagher-Thompson, Rose, and Lovett [40], social support that allows caregivers to engage in social interaction for fun and recreation is the most important in decreasing perceived burden. Other forms of social support are informational and emotional support and tangible assistance. Respite care is a special type of social support where social interaction and tangible assistance are combined. Here, the care tasks are temporarily lifted from the informal caregiver’s shoulders in order to alleviate burden. Moreover, respite care should free up time, so caregivers can perform other roles. However, results on the effectiveness of respite care on caregiver burden are heterogeneous [41].

## Methods

For this study, a systematic review was conducted. In this section, the methodology is described, consisting of a database search strategy, inclusion criteria, and a selection process. The first author (NL) developed the search strategy and performed the first search, which initially resulted in 13 articles. The search strategy (databases, search terms, and inclusion/exclusion criteria) was repeated independently by the second author (JvB). Differences were resolved by discussion, resulting in the addition of four more articles.

### Database search strategy

Three databases were used for the literature search: Medline, PsycINFO, and Scopus. Medline and PsycINFO were searched simultaneously via EBSCOhost. In order to achieve relevant search hits, a field code (namely IT) was used in all databases. It was decided to ensure that the terms caregiver, carer, or caregiving AND burden, stress, strain, burnout, or overstrained were present in the title of the search hits. To narrow down the search results further, the articles had to be written in English, and published from 2013 onwards up to 31st of January 2019. A detailed overview of the search strategy can be found in Table 1. The multiple database search provided a total of 528 articles, after removing duplicates. The articles were then stored in an EndNote X8 database.

**Table 1:** Search strategy (as conducted on 31st of January 2019)

Database	Search strategy		Hits
Medline & PsycINFO via EBSCOhost	#1	TI caregiver OR TI carer OR TI caregiving	37,659
	#2	Informal OR family OR spouse OR partner OR relative	2,756,180
	#3	TI burden OR TI stress OR TI strain OR TI burnout OR TI overstrained	450,956
	#4	Determinants OR factors OR causes OR reasons	6,905,652
	#5	#1 AND #2 AND #3 AND #4	1601
	**#6**	**Limit #5 to English AND from 2013 onwards**	**659**
Scopus	#1	TI caregiver OR TI carer OR TI caregiving	28,197
	#2	Informal OR family OR spouse OR partner OR relative	4,048,355
	#3	TI burden OR TI stress OR TI strain OR TI burnout OR TI overstrained	744,861
	#4	Determinants OR factors OR causes OR reasons	10,995,149
	#5	#1 AND #2 AND #3 AND #4	1049
	**#6**	**Limit #5 to English AND from 2013 onwards**	**459**
Overview		Total number combined records	1.118
		**Records after removing duplicates**	**528**

### Inclusion criteria

In order to reach a final selection of articles, multiple inclusion criteria were established. Firstly, (i) caregiver burden needed to be a key concept in the study and had (ii) to include the multidimensionality of caregiver burden, corresponding with the theoretical framework of this research (i.e. include both objective burden measures *(such as time spent on caregiving, the nature and number of tasks)* and subjective burden measures *(such as caregiver perceptions of the care demands and their consequences, such as negative mood states and anxiety)*). In order to focus the literature review on the context of caregiving at home, (iii) studies with institutionalized or hospitalised patients (i.e. inpatients), were excluded. Moreover, (iv) the patients needed to be still alive during the research period in order to focus the research on caregiver burden during caregiving (i.e. no retrospective orientation). Next, (v) only studies that examined associations between informal care and some measure of burden or stress were included, and (vi) articles had to report empirical research with a minimum of 100 respondents to ensure that statistical relationships and differences were relevant and representative for a whole population. Rather than calculating statistical power using minimum effect sizes of interest, this cut-off point was based on the reasoning that multivariate statistical techniques require at least 15–20 observations per predictor variable [42], and that the number of predictor variables including demographics is at least 6.

Furthermore, (vii) the research had to be conducted in a Western country, because the presumed societal and cultural differences between Western and non-Western countries might play a role in informal care. Lastly, the articles had (viii) to be published in a peer-reviewed journal.

### Selection process

**Fig. 2:**
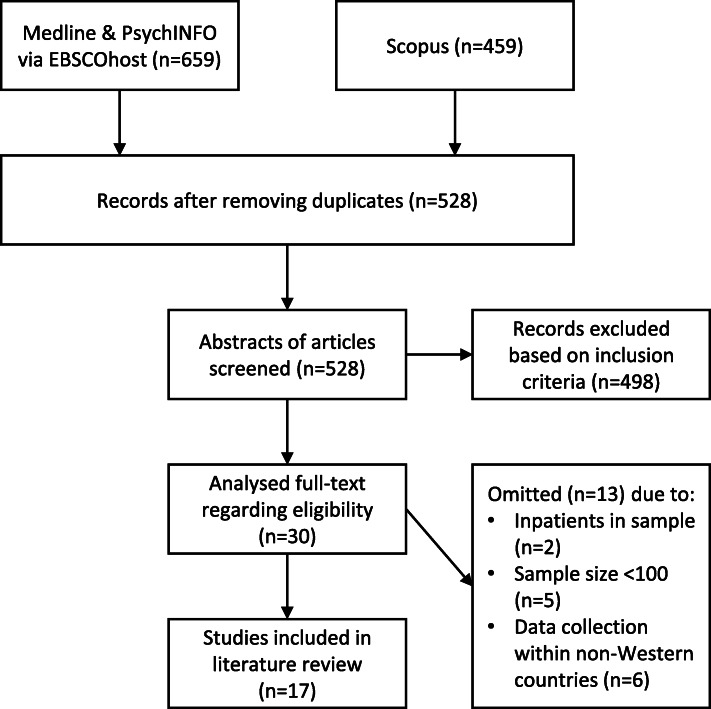
Flow diagram of the selection process

As a first step of the selection process, the abstracts of the articles were screened on the established inclusion criteria. This resulted in a preliminary selection of 30 articles. Then, the full texts of this selection were critically analysed based on the same inclusion criteria. From this, another 13 articles were omitted, resulting in a final selection of 17 articles for the current literature review (Fig. 2). An overview of the included articles can be found in Additional file 1.

## Results

Of the 17 included studies, 14 published cross-sectional studies and three reported longitudinal studies. Thirteen studies measured caregiver burden via validated research instruments. Four studies used only one instrument: the Zarit Burden Interview (ZBI). This specific instrument was used in every paper that reported on caregiver burden in dementia care. An exception was Laporte Uribe’s article [43], which used BIZA-D as an alternative to ZBI. A descriptive table with all included studies can be found in Additional file 1. In this section, the results regarding stressors, moderators, miscellaneous factors, and background characteristics are discussed.

### General stressors

The three longitudinal studies all found that the duration of caregiving and the patient’s functional status were the strongest determinants of burden [44–46]. In each longitudinal study, caregiver burden increased non-linearly over time, as perceived burden increased at a steeper rate as time progressed. This shows that duration is an important factor. Guerriere et al. [44] argued that this might be caused by the progression of the care recipient’s disease. However, it can also be hypothesised that duration has a more direct effect on caregiver burden, independent of disease progression; the burden becomes heavier the longer caring tasks are performed. This, then, could be explained via H3, where duration is expected to increase role overload.

Moreover, these three longitudinal studies as well as six of the 14 cross-sectional studies considered the patient’s functional status in terms of dependency level in activities of daily living (henceforth shortened to ADL) as one of the strongest predictors of perceived caregiver burden [43–51]. This is consistent with the ASM, where ADL dependency is considered a general stressor. The influence of dependency level could be explained via an increase in both the intensity of caregiving and the number of caregiving tasks required, leading to higher burden [44, 47]. Lethin et al. [45] linked ADL dependency in their longitudinal study specifically with increased need for supervision hours in the case of dementia care, providing support for H2.

In contrast to the duration of caregiving, the role of hours spent on caregiving per week or month showed mixed results. De Almeida Mello et al.’s [48] and Blanthorn et al.’s [52] cross-sectional research and Lethin et al.’s [45] and Guerriere et al.’s [44] longitudinal studies considered hours spent on caregiving a determinant of caregiver burden. However, Riffin et al.’s cross-sectional study [51] reported that time spent is no longer associated with caregiver burden after accounting for the number of caregiving tasks. According to them, the allocation of additional responsibilities may be more taxing on the informal carer than the time demand. From this, it can at least be hypothesised that more indirect factors, such as the number of tasks, influence caregiver burden and the perceived time demands.

Another factor that influenced informal caregiver burden is the specific illness of the patient. This is confirmed by four studies that claim that illnesses such as dementia, solid tumours, physical disabilities, or the presence of comorbidities are positively associated with burden [46, 53]. This confirms the place of specific illness as a general stressor in the ASM and supports H1, i.e. general stressors (such as specific illness) have direct effects on perceived burden in addition to the indirect effects identified in H2, H3, and H4.

Finally, the care recipient’s mental state – behaviour and cognitive capacity – appeared to be relevant [43, 45, 46, 54]. This matches with the ASM, where these factors are labelled as general stressors, and again confirms the direct effects of behaviour and cognitive capacity on burden as formulated in H1. Both Ransmayr et al.’s [46] and Lethin et al.’s [45] longitudinal studies and Laporte Uribe et al.’s cross-sectional studies [43] incorporated patient’s behavioural problems such as agitation and aggression in their research, and this proved to be positively related to caregiver burden. These factors seem especially relevant in the context of dementia care, where such behavioural problems occur frequently.

Besides behaviour, three articles, both longitudinal and cross-sectional, reported on care recipients’ cognitive capacity [45, 48, 55]. From the research in these articles, it becomes apparent that the patient’s cognitive status indeed has predictive value for caregiver burden; reduced cognitive function in the recipient leads to higher burden.

In conclusion, all general stressors included in the ASM have direct effects on caregiver burden, supporting H1. Studies on dependency level as a stressor provided indirect support for H2, in addition to one study that specifically confirmed that dependency level translated into an estimation of the needed supervision hours.

### Role and intrapsychic stressors

Ransmayr et al. [46] found in their longitudinal study that burden also builds over time as a result of restrictions on caregivers’ time for other activities and psychological distress. This could be linked to role theory*,* entailing both role conflict, which most likely is the case when less time is available for other activities, and role captivity or role overload, referring to psychological distress.

The relevance of role conflict is confirmed by three cross-sectional studies that firstly showed the influence of job–caregiving conflicts, which are incorporated as one type of role conflict in the ASM [48, 50, 56]. Their research showed not only that informal carers who combine work and care experience the highest burden [56], but also that a higher ADL dependency in combination with employment is positively related to caregiver stress [50]. Secondly, De Almeida Mello et al.’s cross-sectional study [48] confirmed that both job–caregiving conflict and revealed family conflict positively influence caregiver burden, which is also a type of role conflict.

Taken together, these studies support H3 and H4; informal care may lead to both role conflict and role overload, and these, in turn, may result in additional intrapsychic stressors. Moreover, the cross-sectional studies provide support for H1, as role conflict directly affects burden.

### Social support as moderator

Five studies, both longitudinal and cross-sectional, included social support in their research [43, 44, 50, 51, 55]. It was found that a higher quality of support, both formal and informal, for the caregiver is associated with lower burden, whereas unmet needs for health and social services contribute to caregiver burden [55]. Moreover, Hsu et al.’s cross-sectional research [50] referred to the importance of social support aimed at the patient leading to lower caregiver burden. From this, it can be deduced that it is important to ensure that both the caregiver and the recipient feel well supported both by the direct environment and by available professionals. Surprisingly, the articles did not explain, either in theory or via empirical testing, the exact mechanisms that cause social support to affect caregiver burden. However, the fact that social support at least correlates negatively with burden partly supports H5 about the moderating effect of social support on the stressor–burden relationship. No studies addressed the effects of specific coping strategies on care burden.

The specific role of respite services showed some mixed results at first glance. Guerriere et al.’s longitudinal research [44] found that a patient’s number of hospice days were negatively related to caregiver burden; the more days a patient spent in a hospice instead of at home, the lower the burden for the informal caregiver. Hospices are often used as a type of social support or respite service, where patients are admitted during the day or for several days in the week to alleviate the carer’s care responsibilities. However, Riffin et al.’s cross-sectional research [51] showed a positive relationship between using respite services and burden. This seems like contradictory evidence, but it should be stated that causality cannot be determined from cross-sectional research. Therefore, it could be argued that a positive relationship is found consequent to the progression of the disease, which indirectly influences the need for, and use of, respite services because of the intensity of caring. These potential effects of the general stressors on the moderators have not been hypothesised.

### Miscellaneous factors

De Almeida Mello et al.’s cross-sectional research [48] was especially interesting for the current literature review, as they based their empirical research on Bastawrous’ [26] recommendations, which were also used for the ASM in this review. They used the factors from Pearlin et al.’s stress process model [29] as input for their research, in addition to potential determinants from role theory. Additional factors that appeared relevant in their research, besides the already mentioned determinants, were depressive symptoms, previous admissions to nursing homes, risk of falls, cohabitation, and an adult–child relationship between the caregiver and the care recipient. These all had a positive relationship with caregiver burden; this can be interpreted as support for H4. Lethin et al. [45] and Juntunen et al. [55] confirmed that (risk of) depression correlates with caregiver burden, especially among men in Juntunen et al.’s research [55]. However, whether cohabitation and the relationship between caregiver and patient are significant determinants of caregiver burden is disputed in the included articles.

In the cross-sectional and longitudinal studies of Braich et al. [47], Guerriere et al. [44], and Hsu et al. [50], the relationship between caregiver and care recipient appeared irrelevant. In contrast, Riffin et al. [51] and Laporte Uribe et al. [43] in their cross-sectional studies found that an adult–child relationship leads to higher burden. These contradictory results could be explained by the presence of different types of burden. Two cross-sectional studies identified different types of burden via exploratory factor analysis [56, 57]. According to them, caregivers experience different types of burden depending on the specific kinship role. This is in line with, for instance, Juntunen et al.’s cross-sectional research [55] where differences were found in the determinants of caregiver burden for spouses, daughters, and mothers of the care recipient. Therefore, the relationship type between caregiver and recipient can be considered relevant and would most likely be a valuable addition to the ASM.

Lastly, Guerriere et al.’s longitudinal research [44] found living arrangements, such as cohabitation, to be insignificant. However, Ransmayr et al. [46] in their longitudinal research state that the physical proximity of the patient correlates positively with caregiver burden. Here, results remain inconclusive, and unambiguous conclusions on living distance cannot be drawn.

### Background characteristics

Finally, some interesting background characteristics were researched in the selected articles. Seven of the 17 articles indicated that being a female caregiver correlates positively with caregiver burden. In other words, female caregivers experience more subjective burden than male caregivers. Whether the sex of the patient and the age of the caregiver and the patient are also relevant cannot be concluded, as results were inconclusive. However, the overall health, well-being, and quality of life of the caregiver proved to be important. For instance, Riffin et al.’s research [51] found that caregivers in poor health or with anxiety symptoms experience higher informal caregiver burden.

## Discussion

The aim of the present review was to update and synthesize the literature on the common determinants of the caregiver burden, to ultimately ensure the future continuation of informal care in the home context, and improve or sustain the quality of life of caregivers and patients alike. This systematic review tests, and provides evidence for, the ASM in Western countries.

In the studies included in the literature review, all of the determinants incorporated in the ASM were found to have direct effects on caregiver burden, providing strong support for H1. The most important predictors of caregiver burden were the duration of caregiving and the patient’s dependency level. The longer someone had to provide informal care or the more dependent the patient became, the higher the perceived burden. Besides physical dependency, the recipient’s mental state in terms of behavioural problems and cognitive capacity was also a determinant of dependency level, and these were positively related to caregiver burden. Some specific illnesses, such as dementia or solid tumours, also led to a higher burden.

Even though duration of caregiving proved to be an important predictor of caregiver burden, the support for H2 was more mixed, partly because time spent on caregiving appeared to be an ambiguous construct; studies reported either the total duration of caregiving or the hours spent per week or month. Only one study found evidence that the stressor, dependency level, resulted in an estimate of the number of care hours to be spent. Even though both duration and time spent were positively related to caregiver burden, the exact reasons for these effects are not fully clarified. Duration of caregiving would be a valuable addition to the ASM.

Potential explanations for the effects of duration of, and time spent on, caregiving were progression of the disease, role captivity, or role conflicts, indirectly supporting H3. In addition, the presence of role conflicts or role captivity also increased informal caregiver burden. However, the articles did not investigate the causal relationship between role stressors and intrapsychic stressors, so no definitive answer can be given regarding H4. Social support seemed to lower the perceived burden thanks to its moderating role on the relationship between stressors and perceived burden. No studies reported on the effect of coping strategies on caregiver burden; therefore, H5 is only partially supported. In terms of background characteristics, female caregivers experienced more stress over time. In addition, different kinship roles led to different types of caregiver burden. Therefore, the relationship type between caregiver and care recipient should be added to the ASM. Lastly, it should be noted that many findings were discussed as covariates in the articles, but causal relationships could not be derived, as the studies did not determine causality both theoretically and via empirical testing.

### Academic relevance

Adelman et al. [21] published a clinical review about determinants (which they termed risk factors) of caregiver burden in 2014. When comparing the outcomes of the current literature review with that clinical review of a few differences stand out (see Table 2 for an overview). First, as Adelman et al. provide risk factors for caregiver burden, they only address hypothesis 1 from the ASM: a direct effect of stressors on caregiver burden. The current review also found support for these direct relations, but also found some support for an indirect (i.e. mediated) effect number of hours spent on caregiving (H2) and through role stressors (H3). In addition, support was found for a moderating effect of social support, on the relation between general stressors and caregiver burden (H5).

**Table 2:** Synthesis of determinants of caregiver burden: comparison between Adelman et al. (2014) [21] and the current review

Adelman et al. (2014)^a^ [21]			Current review		
Hypothesis ASM^b^ (support found, no support found)	Categorisation	Risk Factors	Determinants	Categorisation (ASM)^b^	Hypothesis ASM^b^ (support found, no support found)
H1 (direct relation)	Demographics	Female sex	Female sex	Background characteristic	H1 (direct relation)
		Low education	–		
		Cohabitation with care recipient	Cohabitation/Living distance	Miscellaneous factor	Inconclusive
H1 (direct relation)	Psychosocial	Coping strategies	Coping strategies	Moderators	H1 (direct relation) H5 partially supported: no moderation of coping strategies
		–	Social Support		H5 partially supported: moderation of social support
		Depression and depressive symptoms	Global (self-esteem) Situational (loss of self, competence)	Intrapsychic stressors	H1 (direct relation) H4 (mediation) not supported
		Perceived patient distress	–		
H1 (direct relation)	Caregiving context	Social isolation and decreased social activity	Role conflict (Restriction of social life)	Role stressors	H1 (direct relation) H3 (mediator between duration of caregiving and caregiver burden)
		Inability to continue regular employment	Role overload		
		Financial stress	–		
		Lack of choice	–		
		Caregiving time and effort	Duration of caregiving	General stressors	H1 (direct relation) H3 (mediated by role stressors)
		–	Specific Illness		
		–	Dependency levels, both physically as mentally		
		–	Care demands		
		–	Problematic behavior		

^a^Refers to risk factors of caregiver burden in Table 1 “The Epidemiology of Caregiver Burden” p1054 Adelman et al. (2014) [21]

^b^The Adapted Stress Model (ASM) is based on stress theories, notably the transactional model of stress and coping (Lazarus, 1984) [28] and the stress process model (Pearlin, 1990) [29], as well as role theory (Biddle, 1986) [30]

Next, Adelman et al. [21] focus on risk factors related to the caregiver (e.g., demographics, such as female sex and low education), and psychosocial factors (e.g., social isolation and decreased social activity). Although attention is also paid to the caregiving context (comprising risk factors such as caregiving time, and lack of choice of being a caregiver), these risk factors seem predominantly focussed on individual caregiver characteristics.

Adding to those insights, the current review found that care recipients’ characteristics are also related to caregiver burden. Specifically, dementia, solid tumours, physical disabilities and the presence of comorbidity were associated with higher burden. The associations of these specific illnesses with caregiver burden could partly be explained by the different levels of care recipients’ mental and/or physical dependency resulting from these illnesses, as these dependency levels have also been found to be relevant determinants of caregiver burden. This again confirms the importance of the specific illness as a general stressor of caregiver burden.

### Practical implications

Based on this literature review, recommendations can be made for future interventions. Firstly, it is important to focus on the specific carers most at risk of caregiver burden, as research has shown that being a female informal caregiver, providing dementia care, and being a child’s caregiver predict higher burden. Besides specific interventions for these groups, it appears that increased involvement of men in informal care would provide an opportunity to relieve the burden for female caregivers. The current social norm holds that women take up more caring tasks than men; however, such role expectations threaten the sustainability of informal care in light of rising care demands in the future.

Secondly, interventions could benefit from adaptation to the course of specific illnesses, as it appears that burden increases non-linearly over time. For example, it could be determined at which junctures during the illness trajectory interventions would be most needed to support caregivers. This could involve interventions on different levels, such as interventions aimed at the individual, where the focus could be on increasing the patient’s independence both mentally and physically, and interventions aimed at the organisations involved in the caregiving trajectory.

### Future research

Some interesting future research opportunities are indicated by this literature review, the outcomes of which could be used to optimise interventions aimed at relieving informal caregiver burden. First and foremost, experimental designs are needed to determine the causality between determinants. Specifically, the effects of different types of respite care could be tested experimentally regarding their effectiveness in relieving care burden.

Moreover, this research domain would benefit from more longitudinal studies, enabling trend analyses regarding the trajectory of informal caregiver burden over time. As the concept of time cuts straight through both the primary and the secondary stress appraisals, and contains both objective and subjective elements, time warrants further attention. For instance, it influences not only the appraisal of the stressful event of informal caring, but also the perceived hours available for other roles.

Lastly, during the selection process, it was noted that a large proportion of the research appeared to have been carried out in Asian countries, which have collectivistic cultures, and these studies were excluded from the current review. It would be interesting to conduct a comparative study between the Western and the Asian world in order to determine the relevance of cultural expectations for informal caregiver burden.

### Strengths and limitations

Some limitations of this research should be noted. Firstly, most of the articles included in the systematic review consisted of cross-sectional research. This may be explained by the search strategy, in which we did not specify research designs (such as an RCT). Therefore, causality between the different concepts could not be determined properly. Due to this, the actual process or framework (via indirect effects) cannot be validated.

Secondly, a methodological limitation is the selection criterium for studies having at least a sample size of 100 subjects, a cut-off point that was somewhat arbitrary. Larger sample sizes would have increased statistical power (avoiding type I error). However, since some patient groups (and therefore also the caregivers groups) are relatively small, potentially relevant relationships may have been missed (increasing type II error) [58].

Furthermore, this study only included quantitative studies. More rich and in-depth insights on how caregiver burden comes about, could have been established by including qualitative studies. Thereby, the current review only tests the ASM, but does not expand it.

Other limitations are related to the search strategy. Firstly, using general search terms (i.e. not illness-specific) was in line with the aim of the study to identify common causes of caregiver burden. However, we might have missed information on common causes identified in studies that were reporting on caregiver burden for a specific illness. Secondly, the search terms were informed by the Adapted Stress Model, which formed the theoretical underpinning for this review study. By making this choice, we may have missed articles referring to for example ‘associations’ instead of determinants, factors, causes or reasons. In addition, we may have missed articles that did not include the terms caregiver, carer or caregiving AND burden, stress, strain, burnout or overstrained in the title. Finally, using 2013 as the lower date limit in the search strategy can be considered an arbitrary choice. However, this was done in order to increase the relevance of our systematic review, since more recent studies might already have been affected by ongoing policy reforms that aim to lessen the burden on formal care, through stimulating informal care in the home context.

Lastly, the ASM, used as underpinning for this review, also has its limitations. For example, it does not take into account (personal) resources (such as self-efficacy etc.) which might also be relevant in predicting caregiver burden. Considering the multidimensionality of caregiver burden and its outcomes, this could have resulted in overlooking some determinants or working mechanisms.

However, overall, this systematic review also has certain strengths. In the vast and diverse array of articles available on the topic of informal caregiver burden, a focused selection has been made with a systematic and transparent search strategy. This strategy comprised of selecting only studies with large samples (*n* > 100), as those studies have larger explanatory (statistical) power than studies involving smaller samples. Moreover, this literature review has a strong theoretical framework, which already identified most of the determinants later found during the review. The reviewed studies thus provided more insights into if and how these determinants affect caregiver burden.

## Conclusions

According to the present review, all determinants as described in the Adapted Stress Model have direct influences on caregiver burden. The strongest determinants of caregiver burden are the duration of caregiving, and the patient’s dependency level. In addition, the patient’s mental state, in terms of behavioural problems and cognitive capacity, determines dependency level, and thus care burden. Therefore, specific interventions should be designed for carers most at risk, which focus on increasing the patient’s physical and mental independence. Interventions to relieve burden need to be adapted to the illness trajectory of specific diseases and corresponding needs for social support for both the recipient and the caregiver. Finally, changing role expectations, leading to men being more involved, could reduce the disproportionately high burden for women.

## Supplementary information

**Additional file 1.** Output literature review

## Data Availability

All data generated or analysed during this study are included in this published article [and its supplementary information files].

## Abbreviations

ADL: Activities of daily living ASM Adapted stress model ZBI Zarit Burden Interview
