# Supplementary material for: Determinants of overburdening among informal carers: a systematic review
Source: BMC Geriatr. 2020 Aug 26;20:304. doi: 10.1186/s12877-020-01708-3 (PMC7448315; doi:10.1186/s12877-020-01708-3)
Supplement: Supplementary file 1 — Additional file 1. Output literature review [file 12877_2020_1708_MOESM1_ESM.docx]

# Appendix 1 Output literature review

| Study | Design | Outcome measures | Results / Conclusions |
| --- | --- | --- | --- |
| Blanthorn-Hazell et al. (2018) | Cross-sectional study (n=297) of paired informal caregivers and their patients with bipolar disorder and schizophrenia, Europe: UK, Germany, Spain | Demographics, disease characteristics, burden of agitation, awareness of agitation episodes, patient symptoms, coping strategies, Involvement Evaluation Questionnaire for caregiver (tension, supervision, worrying, urging) | The number of hours of care provided and patient behaviour increase the perceived burden. Spanish informal caregivers experienced higher caregiver burden, especially in ‘worrying’ domain. |
| Braich et al. (2016) | Cross-sectional study (n=486) of informal carers to legally blind family members, New York State, USA | Care burden (time-dependent, emotional, existential, physical, service-related, personal estimate of overall burden), risk of depression, ADL (independence in activities of daily living), participant characteristics (Patient: gender, visual acuity, intensity of caregiving, chronic conditions. Caregiver: gender, age, literacy, relationship to patient, chronic conditions, duration of caregiving, time spent caregiving, required close supervision time of the patient, supplemental caregivers) | Intensity of caregiving and multiple chronic illnesses in caregivers act as covariates in all care burden dimensions, except for service-related burden. Daily hours of close supervision are also considered a covariate of care burden, except for physical burden, existential and service-related. Intensity of caregiving is inversely proportional to independence with ADL. Lastly, females experience more service-related burden. Other characteristics appeared not to be relevant. Level of blindness does not correlate with higher caregiver burden. |
| Cohen et al. (2015) | Exploratory factor analysis (n=1014) of adult children caregivers, USA | Demographic characteristics (age, gender, marital status, educational level, poverty status, relationship with care recipient, presence of minor child in home of caregiver), caregiving burden (health, financial burden, social participation, emotional well-being) | Domains of child caregiver burden identified are positive emotional, negative emotional, social and financial. Intensity of caregiving not incorporated in research. Burden domains consistent over demographic groups. |
| de Almeida Mello et al. (2017) | Cross-sectional study (n=4175) of older persons and their informal caregivers, Belgium | Objective burden (number of hours per week spent caregiving, time spent on supervision), informal caregiver burden via Zarit Burden Interview (personal and role strain), functional dependence (activities of daily living hierarchy (ADLH), instrumental activities of daily living performance (IADLP)), cognitive decline, behavioural problems, incontinence, background characteristics (client’s age, gender of recipient, marital status, living status) | General stressors such as ADL, IADL, depression, cognition, behavioural problems, risk of falls and other factors from Pearlin’s model act as correlates to experienced burden. Cohabitation and time spent providing care, plus determinants from Role theory (caring for others, adult child relationship, leaving part of job) also relevant. Having a female recipient and older aged is negatively correlated with perceived informal caregiver burden. |
| Del-Pino-Casado et al. (2014) | Cross-sectional study (n=140) of family caregivers of older dependents, Spain | Stressors (intensity of care via number of ADL’s, , needs of care recipient, behavioural problems leading from psychological symptoms), coping strategies, subjective burden via CSI, anxiety via HARS. | Subjective burden mediates the relationship between psychological symptoms and anxiety and partially mediates the relationship between dysfunctional coping and anxiety. |
| Flyckt et al. (2015) | Cross-sectional study of patients with psychotic disorders (n=107) and their informal caregivers (n=118), Sweden | Subjective burden of informal care (happiness, health, quality of life), patient characteristics (functional, symptomatic, global functioning, sociodemographic), objective burden (expenses, time spent), caregiver characteristics (health, quality of life, utility, perceived support, work conflict, sociodemographic, living situation), healthcare and community support variables (frequency and type of contact with paramedics, frequency of contact with community) | Level of functioning of patient and health state of caregiver are most potent determinants of subjective burden. Female and elderly caregivers experienced a higher degree of subjective burden. No predominant determinants found for objective burden. Financial burden was related to income of patient and caregiver plus financial public support. Caregiver spent more hours when there was little negative impact of caregiving. |
| Fridman et al. (2017) | Cross-sectional study (n=2326) of caregivers of children/adolescents with ADHD, Europe | Comorbidities, ADHD subtypes and severity, treatment adherence, caregiver burden (work, social life, family life, worry/stress) | Child/adolescent comorbidities and ADHD severity act as correlates to all burden concepts, especially with caregivers’ work and social-related outcomes. This relation strengthens with an increase in severity or number of comorbidities. Improved medication adherence is associated with reduced caregiver burden for the outcomes of altered work, worry about perceptions regarding their parent role, and avoiding social activity, but not family life or worry/stress. |
| Guerriere et al. (2016) | Longitudinal, prospective cohort study (n=327) of caregivers of patients with malignant neoplasm, Canada | Patient demographic characteristics, caregiver demographic characteristics, patient clinical characteristics (comorbidities, functional status), economic determinants (publicly financed healthcare resources, privately financed resources), caregiver burden | Caregiver burden increased non-linear over time, burden increases as illness progresses. Time-varying covariates were monthly family care-giving time costs (of which time-spent caregiving serves as a proxy), monthly public personal support worker costs (one of stronger predictors, higher costs lead to higher burden probably due to progression disease), emergency department visits, used hospice services (lower burden) and patient functional status (strongest predictor). Time-stable covariates were caregiver gender (female experience higher burden over time), patient gender (higher burden with male recipient), caregiver education (higher education experience higher burden), patient education (higher education experience lower burden). Not related were: patient or caregiver age, marital status of patient or caregiver, relationship of the caregiver to patient, living arrangements. |
| Hsu et al. (2014) | Cross-sectional study (n=100) of caregivers of patients aged >65 years with cancer, USA | Caregiver sociodemographics, relationship to patient, cohabitation, time spent caring, patient sociodemographics, geriatric assessment (patient’s health; cognition, functional status, comorbidity, nutrition, psychological state, and social activity and support), caregiver burden (employment, financial, physical, social, time) | A higher IADL dependency in combination with an employed caregiver results in higher burden. On bivariate analysis, the caregiver being non-Caucasian, employed, aged <66 years and taking care of patient who had unintentional weight loss or solid tumours led to higher burden. Spending more time on providing care, lower functional status of patient, higher IADL dependency, more limitations in social activities of patient due to health, and less social support for patient act as covariates. Caregiver sex, relationship to patient or patient’s emotional state appeared irrelevant. |
| Juntunen et al. (2018) | Cross-sectional study (n=1062) of spouse, adult child and parent caregivers, Finland | Demographic characteristics of caregiver, hours per day and duration of caregiving, use of social and healthcare services, caregiver burden (social, physical, family, financial, role captivity, emotional, coping, satisfaction, quality of social support), caregiver depression, demographic characteristics of patient (physical mobility, cognitive functioning) | Overall, among all three caregiver categories (spouse, daughter and mother), lower level of cognitive function of patient leads to higher burden and higher quality of support (formal and informal) for the caregiver is associated with lower burden. Unmet need for services act as covariate for spouse and mother caregivers. Physical dysfunction is also associated with burden for caregiver mothers, less strong for caregiver daughters. Female spouse caregivers experience greater burden and depression. Signs of depression correlate with burden, especially among men (not mothers). Poor health of caregiver explains burden for all female caregivers. |
| Laporte Uribe et al. (2017) | Cross-sectional study (n=536) of informal caregivers for patients with dementia making use of DCN’s, Germany | Socio-demographics and clinical variables. Burden via BIZA-D standardized questionnaire, consisting of 4 categories: burden due to practical care tasks (i.e. objective burden), subjective burden due to changing behaviours, perceived conflicts of needs, role conflicts. IADL for patient, caregiver health status. | IADL performance and challenging behaviour both negatively impacted perceived burden. Moreover, patient-caregiver relationship and caregiver gender were associated with burden. The presence of an adult-child relationship or being a female caregiver resulted in higher objective and subjective burden. Supporting caregivers resulted in lower burden. |
| Lethin et al. (2018) | Longitudinal, multinational cohort study with a three-month follow-up (n=1223) of dyads of caregivers and care recipients with dementia, Europe | Zarit Burden Interview for caregiver, cognitive functioning of patient, care demands, quality of care | Factors associated with burden are extensive informal care provision (ADL, IADL, time caregiving and supervision), decreased well-being and reduced quality of life for the caregiver and reduced cognition, decreased quality of life, severe neuropsychiatric symptoms (agitation and aggression), IADL dependency and depression in the patient and caregivers’ negative experience of quality of care. |
| Polenick & Martire (2013) | Cross-sectional study (n=212) of spouses and adult child caregivers of older adults, USA | Caregiver characteristics, character attributions (caregiver perceptions of the patient as lazy or lacking willpower or discipline), intention attributes (beliefs of symptoms as manipulative and deliberate behaviour), controllability (idea of patient being in control of behaviour). General caregiving burden (impact on health, social life, family responsibilities etc., via 22-item burden interview) Depression-specific burden via Mood Disorder Burden Index. | Character attributions predicted both general and depression-specific burden, intention attributes predicted general burden. Controlabillity attributions did not predict either type of burden. |
| Ransmayr et al. (2018) | Multicentred longitudinal study (n=585) of persons caring mostly for Alzheimer’s disease patients, Austria | Demographic and clinical characteristics of patients, caregiver burden interview (frequency of limitations in personal and social life, health strain, feelings of insufficiency and guilt), caregiver demographics | Burden correlates positively with NPI (neuropsychiatric inventory, such as behavioural abnormalities), CDR (clinical dementia rating), DS (dependency scale) scores, disability, years of education of patient, and proximity of patient and caregiver sex (female), and negatively with caregiver age. Burden increased over time due to restrictions in time for other activities, psychological distress and increased health problems. |
| Riffin et al. (2018) | Cross-sectional study (n=1742) of family and unpaid caregivers, USA | Caregiver burden (emotional, physical and financial), types of assistance, caregiver and care recipient characteristics (sociodemographic, health, caregiver use of supportive services and availability of help from others) | Higher ADL and IADL dependency, more health management tasks and more health system logistics lead to higher burden, as well as female caregivers, adult child caregivers, using respite care, dementia and caregivers in poor health or with anxiety symptoms. Hours spent caregiver no longer associated with burden after accounting for number of caregiving tasks (provision of additional responsibilities may be more taxing than the time demand). |
| Unson et al. (2016) | Cross-sectional study (n=169) of informal caregivers, New England, USA | Zarit Burden interview (health, finances, social life, interpersonal relationships), recipient characteristics, caregiving arrangements, caregiver sociodemographic characteristics, nature of care (kinship roles) | Five factors of burden: personal strain/loss, uncertainty/inadequacy, social strain/embarrassment, anger and dependency. Caregivers experience different types of burden depending on gender, kinship roles and level of involvement in care. Solo caregivers and younger caregivers, who are more likely to combine work and caregiving, experience highest burden. |
